# Increased AHR Transcripts Correlate With Pro-inflammatory T-Helper Lymphocytes Polarization in Both Metabolically Healthy Obesity and Type 2 Diabetic Patients

**DOI:** 10.3389/fimmu.2020.01644

**Published:** 2020-07-30

**Authors:** Ru-xing Zhao, Qin He, Sha Sha, Jia Song, Jun Qin, Peng Liu, Yu-jing Sun, Lei Sun, Xin-guo Hou, Li Chen

**Affiliations:** ^1^Department of Endocrinology, Qilu Hospital of Shandong University, Jinan, China; ^2^Institute of Endocrine and Metabolic Diseases of Shandong University, Jinan, China; ^3^Jinan Clinical Research Center for Endocrine and Metabolic Diseases, Jinan, China; ^4^Department of Internal Medicine, Affiliated Hospital of Shandong Huayuan Mining Co. Ltd, Taian, China

**Keywords:** aryl hydrocarbon receptor, metabolically healthy obesity, type 2 diabetes, CD4+ T cells, metabolic inflammation, gut microbiota

## Abstract

Aryl hydrocarbon receptor (AHR) is a ligand-activated transcription factor whose transcription activity is regulated by small compounds provided by diet, xenobiotics, and metabolism. It has been proven to be involved in energy homeostasis and inflammation in most recent years. Epidemiologically, exposure to xenobiotic AHR ligands contributes to obesity and type 2 diabetes (T2D). AHR is also the critical transcription factor determining the lineage commitment of pro-inflammatory Th17 and Th22 cells from naïve CD4+ T lymphocytes. It has been well-illustrated in animal models that IL-22, the major effector cytokine of Th17 and Th22 cells, played a major role in the interaction of metabolism and gut microbiota. But there were still missing links between gut microbiota, IL-22, and metabolism in humans. Our previous findings indicated that elevated circulating levels of IL-22 and frequencies of Th22 cells were associated with insulin resistance in both patients with obesity and T2D. Additionally, the hyperactive Th17 and Th22 cells phenotype also correlate with islets β-cell dysfunction in T2D. In this study, we made efforts to determine AHR expressions in peripheral blood mononuclear cells (PBMCs) from patients with T2D and metabolically healthy obesity (MHO). Correlation analyses were conducted to assess the possible link between AHR and the metabolic and inflammatory context. We revealed that mRNA expression of AHR was up-regulated and correlated with the percentage of Th17, Th22 as well as Th1 cells. Elevated plasma levels of IL-22 and IL-17 also correlated with increased AHR transcripts in PBMCs from both MHO and T2D patients. The transcription factor AHR may thus have a plausible role in the interaction between metabolism and pro-inflammatory status of patients in the development of obesity and T2D.

## Introduction

The prevalence of obesity, in parallel with type 2 diabetes (T2D), has expanded immensely in recent years and has risen as a global epidemic concern (1). Epidemiological and clinical insights offer substantive clues in our understanding of molecular pathways and physiologic systems underlying the regulation of metabolic balance (2). Beside the long list of metabolic comorbidities, obesity, and T2D have also been linked to chronic low-grade systemic inflammation (meta-inflammation), which is postulated to be causative in the development of both insulin resistance and progression to diabetic complications (3–6). The study of how metabolic stress impact the regulation of immune homeostasis and how the immune imbalance triggers the meta-inflammation in return are now topics of intensive investigation. Previous works revealed that T cell subsets were important regulators of meta-inflammation in both animal models and patients with obesity and T2D (7). These studies identified an significant elevation in the Th17 and Th1 subsets together with a decrease in the Treg subset (3, 4). Pro-inflammatory polarization of T helper (Th) lymphocytes, with hyperactivated Interferon-γ (IFN-γ) and interleukin-17 (IL-17) producing Th subsets (Th1 and Th17) and/or impaired regulatory T cells (Tregs), could directly trigger the activation of the innate immunity, and subsequently lead to meta-inflammation and insulin resistance in obesity and T2D (8). We as well as other independent teams have even identified Th22 as a novel potent participant in the development of obesity and diabetes (9–11). Our previous finding indicated that elevated serum IL-22 levels and Th22 frequencies were associated with insulin resistance in both obesity and T2D patients (9, 12). Additionally, the hyperactive Th17 and Th22 cell phenotype also correlate with islets β-cell dysfunction in T2D (9). The notable correlation of pro-inflammatory Th subsets with clinical parameters in patients implicate that the immunologic disturbance may play more determinant roles in both insulin resistance and β-cells dysfunction (9, 12–14). However, up until recently, the specific immunologic sensors involved in response to metabolic stress to produce such a state of immunologic disturbance were not identified.

The aryl hydrocarbon receptor (AHR) is an evolutionarily conservative ligand-activated transcription factor who was first identified by its role in modulating the organism's response to xenobiotics (e.g., toxicants such as polycyclic aromatic hydrocarbons and other environmental pollutants), and physiological molecules such as dietary indoles (15). It can be bound and activated by a wide range of small compounds provided by intrinsic metabolites, commensal microbiota, diet, and the environment (16). Upon ligand binding, AHR translocates to the nucleus, prompts protein–protein interactions with type 2 basic helix–loop–helix PER-ARNT-SIM (bHLH-PAS) proteins (i.e., Arnt) and regulates the subsequent transcriptional activity involved in detoxification (i.e., Cyp1a1), NF-κB regulation, and immune modulation (17). In the past decades, AHR has also been increasingly established as an important modulator of disease, especially for its emerging role in regulating immune responses and inflammation (18). AHR has been proved to be the determinant transcription factor driving the lineage commitment of Th22 from naïve CD4+ T cells (19). It has also been shown to regulate the developmental programs of Tregs and Th17 cells (20). Moreover, there is a reciprocal relationship between the activated AHR and the molecular circadian clock activation (21). Epidemiological evidence also revealed that exposure to xenobiotic AHR ligands such as polycyclic aromatic hydrocarbons could contribute to the incidence of obesity and T2D (22, 23). Taken together, the multiplicity of AHR signaling may shed light on the understanding of the integrated network of environmental factors, immune response, and energy metabolism.

Up to now, there is a growing body of evidence concerning the role of AHR signaling in obesity and T2D (21, 24). It has been well-illustrated in animal models that IL-22, the major effector cytokine of Th17,Th22, and type 3 innate lymphoid cells (ILC3), plays a critical role in the interaction of gut microbiota, mucosal immunity and metabolism (25–27). Our previous data add evidence to the pro-inflammatory Th polarization in obese and T2D patients (9). However, to our knowledge, there was no study present concerning the expression of the AHR gene in patients with obesity or T2D. Since Th17 lineage, driven by Retinoic acid receptor related orphan receptor gamma (RORC), represents another source of IL-22, we made efforts to investigate the expression of the transcription factor AHR, as well as RORC, in peripheral blood mononuclear cells (PBMCs) from patients with T2D and metabolically healthy obesity (MHO) subjects. Correlation analyses were further conducted to assess the possible link between the key transcription factors and the metabolic and inflammatory parameters in different groups of subjects.

## Materials and Methods

### Subjects

Enrollment of participants took place from April, 2013 to December, 2015 in Qilu Hospital, Shandong University, China. Participants were generally arranged into three groups. The T2D group was selected from clinically definite T2D inpatients in the Department of Endocrinology & Metabolism, Qilu Hospital. The age of the disease onset was above 35 year-old for all participants. Serum levels of autoantibodies including anti-islet cell antibody, anti-insulin antibody and anti-glutamic acid decarboxylase antibody as well as anti-thyroid peroxidases antibody were all negative. Detailed clinical records were kept for all patient including the disease history, physical, and laboratory findings, to exclude Type 1 diabetes and other autoimmune disorders (e.g., autoimmune thyroid disease). Two control groups were selected. The first included healthy subjects with a normal Body Mass Index (BMI) referred to as the healthy control (CTL) (BMI: 19–23.9 kg/m^2^), and the second included subjects that fell into the category of metabolically healthy obesity [MHO; which was defined as patients with a BMI ≥ 30 kg/m^2^ and normal glucose tolerance and lipid profiles as previously described (9)]. Subjects from both groups were selected from age-matched healthy volunteers from local neighborhood communities and hospital staff. All subjects from both control groups were given a general medical examination including history of disease, physical, and laboratory examinations, to exclude hyperglycemia, hyperlipidemia, hypertension, and diagnosed autoimmune disorders. A total number of 80 participants, including twenty healthy control (CTL), thirty MHO and thirty T2D patients, were consecutively enrolled to guarantee the sufficient power (>0.80) for the study. Exclusion criteria included any clues of other autoimmune diseases, acute, and chronic infections (within the past 4 weeks), fever of unknown origin, tumors, or significant elevation in erythrocyte sedimentation rate etc. Participants should not use immunomodulatory or immunosuppressive agents in the past 12 months prior to sampling. Our research was approved by the Medical Ethical Committee of Qilu Hospital of Shandong University [No.2013(046)]. The investigation was performed in a blinded manner. Consent forms were obtained from all participants.

### Sample Preparation

Samples for flow cytometric analysis were prepared as previously reported (9). Briefly, fasting peripheral blood were obtained from each donor at seven to nine a.m. Fresh samples were processed within 2 h after collection. Serum or plasma were isolated, and sent for biochemical tests or stored at −80°C for ELISA analysis immediately. Four hundred microliter of the heparinized peripheral blood was diluted with 400 μL of RPMI 1640 (containing 50 ng/mL of phorbol-12-myristate-13-acetate, 2 μg/mL of ionomycin, and 3.4 μg/ml Monensin) and incubated for 4.5 h at 37°C, 5% CO_2_ (all from Sigma, Saint Louis, USA) as described before (9).

The rest of heparinized blood was diluted 1:1 with saline at room temperature and centrifuged on Ficoll-Paque gradients (TBD,Tianjin, China) at 450 g for 20 min. PBMCs from the interface were collected and washed twice in cold phosphate buffer saline, and resuspended using 500 μL TRIzol® Reagent (Invitrogen, CA, USA) for total RNA isolation.

### Flow Cytometric Assay

Stimulated blood samples was further stained to determine the cytokine-producing cells by flow cytometry. Generally, after stimulation, whole blood samples were stained with PerCP-Cy5.5 conjugated anti-human CD4 monoclonal antibodies (Cat No. 85-45-0048-42) at room temperature for 20 min, and then incubated with an equal volume of Fix-perm reagent A for 15 min. After washing, Fix-perm reagent B was added. The sample was subsequently incubated with FITC-conjugated anti-IL-17A monoclonal antibodies (Cat No.85-11-7179-42), PE-conjugated anti-IFN- monoclonal antibodies (Cat No. 85-11-7319-82), and eFluro680-conjugated anti-IL22 monoclonal antibodies (Cat No. 85-50-7229-42) for 20 min at room temperature. Cat No.Cat No.Cat No.Antibodies above were all purchased from eBioscience, CA, USA. Fix-perm reagents were purchased from Invitrogen (CA, USA). Samples were washed and immediately detected by BD AccuriC6 Flow Cytometer. Isotopes were applied as negative controls and. Data were analyzed using FlowJo 7.6. Gating strategy could be referred to as previously reported (9).

### Extraction of Total RNA and Building of cDNA Library by Reverse Transcription

Isolated PBMCs were resuspended using TRIzol® Reagent as described previously. 0.2 ml of chloroform (Sigma-Aldrich, USA) was added to a 500 μL homogenized sample and followed by vigorous vortexing. The samples were centrifuged at 13,000 rpm for 10 min. The total RNA was then extracted from the upper aqueous phase. Total RNA was washed and purified for each sample (purity > 1.75) and synthesized immediately into cDNA using RevertAid™ First Strand cDNA Synthesis Kit (Thermo Scientific, USA) following the instructions. Two microliter of freshly isolated RNA was reverse transcribed in a 20 μL system with 5 × Reaction Buffer, 100 mM dNTP Mix, RevertAid Reverse Transcriptase, oligo(dT)18 primerand RiboLock RNase inhibitor. The reaction was performed in the iCycler thermocycler (Bio-rad, Germany) at 42°C for 1 h and then at 70°C for 5 min.

### Relative Quantification of the Expression of AHR and RORC by Real-Time Quantitative PCR

Real-time quantitative PCR was performed using an ABI Prism 7500 Real-time PCR system (Applied Biosystems, CA, USA). The final volume of 20 μl system contained 1.8 μL of cDNA sample, 10 μl of 2 × SYBR Green Master Mix (Toyobo, Japan), 0.2 μl of Taq polymerase, 7 μl of ddH_2_O and 0.5 μl of the forward and reverse primers, respectively. The sequences of primers were as follows, respectively: AHR Forward 5′-CAA ATC CTT CCA AGC GGC ATA-3′, Reverse 5′-CGC TGA GCC TAA GAA CTG AAA G-3′. RORC Forward 5′-CAA TGG AAG TGG TGC TGG TTA G-3′, Reverse 5′-GGG AGT GGG AGA AGT CAA AGA T-3′; All tests were conducted in triplicates with the following protocol: denaturation at 95°C for 15 s, annealing at 62°C for 15 s, extension at 72°C for 45 s. The PCR primers and protocols were primarily testified by agarose gel electrophoresis to determine product sizes and to confirm that no by-product was formed before real-time quantification. And products were also analyzed by melt curve analysis in the Real-time PCR system. The result was expressed as a ratio relative to the number of glyceraldehyde-3-phosphate dehydrogenase (GAPDH) transcripts used as an internal control. GAPDH was analyzed using the following primers: Forward 5′-AAG GTG AAG GTC GGA GTC AAC-3′, Reverse 5′-GGG GTC ATT GAT GGC AAC AAT A-3′. Relative amounts of gene transcripts normalized to endogenous reference gene was calculated using the comparative Ct method formula 2^−ΔCt^.

### Quantification of Plasma Cytokines by ELISA

The plasma levels of Th1/Th17/Th22 cells predominantly effector cytokines IFN-γ, IL-17A, and IL-22 This was determined with quantitative sandwich enzyme linked immunoassay e as per the manufacturer's recommendations (Multisciences, CN). The minimum detectable concentrations of IFN-γ, IL-17A, IL-22were 0.30, 0.55, 2.56 pg/ml, respectively. Leptin levels were determined using a Leptin Human ELISA Kit (Thermo Scientific, US) with the minimum detectable limit of 15.60 pg/mL. The plasma levels of hypersensitive C-reactive protein (hsCRP) were detected using a hypersensitive latex-enhanced immunoturbidimetric assay by Roche Cobas Integra 800 full-automated analyzer (Roche Diagnostics, North America). The intra-assay and inter-assay coefficients of variation were <5 and 10%, respectively. The lower detecting limit was 0.1 mg/L.

### Statistical Analysis

All data were represented as the mean, ± SD or median (range) according to the data distribution. Differences among 3 groups were determined by one-way ANOVA, and differences between two groups were further determined using the Newman–Keuls multiple comparison tests. Non-normal data were analyzed using the Kruskal–Wallis test and the Dunn test. Correlation was analyzed using Pearson or Spearman correlation test depending on data distribution. Partial correlation test was performed to eliminate interference from age and sex. And multiple linear regression analysis was conducted to further evaluate the relationship between parameters. All tests were conducted by SPSS 18.0 or GraphPad Prism 8.0 system. A *P* < 0.05 was considered statistically significant.

## Results

### Baseline Characteristics of Subjects

Significant differences were found in BMI, fasting insulin, and IL-22 levels within each of the three cohorts as well as between each group. The homeostasis model of assessment for insulin resistance index (HOMA-IR) has been established as an indicator of insulin resistance. Its nature logarithmic value Ln (HOMA-IR) is an even more reliable parameter and was used as a parameter reflecting basal insulin-secreting function of β-cell in this study (28). Although we did not observe significantly higher fasting glucose in MHO participants, there were significant elevations in Ln (HOMA-IR) and serum levels of leptin, insulin, TNFα, IL-22, and hsCRP compared with CTLs. This is inconsistent with previous publications, and the data indicated that obesity, either metabolically healthy or not, was accompanied with hyperinsulinemia, leptinemia, and low-grade inflammation. Ln (HOMA-IR) and serum levels of IL-17, IL-22 were even significantly higher in T2D patients compared with the MHO group, indicating a decompensating inflammatory status. Differences in age, sex, IFN-γ, etc., might show a remarkable tendency but did not show statistical significance in our study. Since we did not ask our patients to stop necessary hypoglycemic treatment (especially insulin preparations and secretagogues) before sampling, the hypoglycemic treatment had potential neglectable influences in insulin levels. The insulin levels in these patients could not reflect the real β cell function level. Therefore, the differences of fasting insulin in T2D patients compared with CTLs and MHOs did not have scientific value. The detailed parameters are demonstrated in Table 1.

**Table 1:** Demographic characteristics of participants.

	**CTL**	**MHO**	**T2D**
Number	20	30	30
BMI (kg/m2)	21.20 ± 1.64	32.62 ± 1.99^*^	24.87 ± 3.87^*^^#^
Age (year)	47.10 ± 8.53	46.07 ± 8.66	50.37 ± 6.92
Sex (female/male)	9/11	13/17	12/18
FBG (mM)	4.76 ± 0.31	5.29 ± 0.41	8.32 ± 3.18^*^^#^
FINS (IU/mL)	5.20 ± 1.20	11.54 ± 4.19^*^	15.81 ± 8.35^*^^#^
Duration of illness (year)	n.a.	n.a.	6.34 ± 3.63
IFN-γ (pg/mL)	0.91 ± 0.89	1.84 ± 2.32	1.58 ± 1.36
IL-17 (pg/mL)	2.11 ± 0.60	2.87 ± 2.07	4.77 ± 1.95^*^^#^
IL-22 (pg/mL)	35.83 ± 2.69	42.78 ± 7.93^*^	51.55 ± 9.73^*^^#^
TNFα (pg/mL)	12.20 (1.50–16.62)	30.38 (18.17–48.65)^*^	49.16 (26.77–91.81)^*^
IL-6 (pg/mL)	7.355 (4.66–14.52)	10.86 (7.76–17.49)	16.95 (7.92–24.01)^*^
Leptin (pg/mL)	3090 (1961–4873)	6078 (5230–9117)^*^	3338 (1979–4877)^#^
hsCRP (mg/L)	0.320 (0.01–1.30)	1.620 (0.70–3.26)^*^	1.255 (0.85–4.36)^*^
Ln(HOMA-IR)	0.1290 ± 0.1363	0.9413 ± 0.3273^*^	1.562 ± 0.5009^*^^#^
Ln(HOMA-β)	4.420 ± 0.3978	4.835 ± 0.4444	4.273 ± 1.094^#^

FBG, Fasting blood glucose; FINS, fasting insulin level; HOMA-IR, The homeostasis model of assessment for insulin resistance index; HOMA-β, The homeostasis model of assessment for β-cell function; n.am not applicable;

* P < 0.05 compared with CTL;

# *P < 0.05 compared with MHO*.

### Elevations of AHR mRNA Expressions Were More Remarkable in PBMCs From T2D Patients

**Figure 1:**
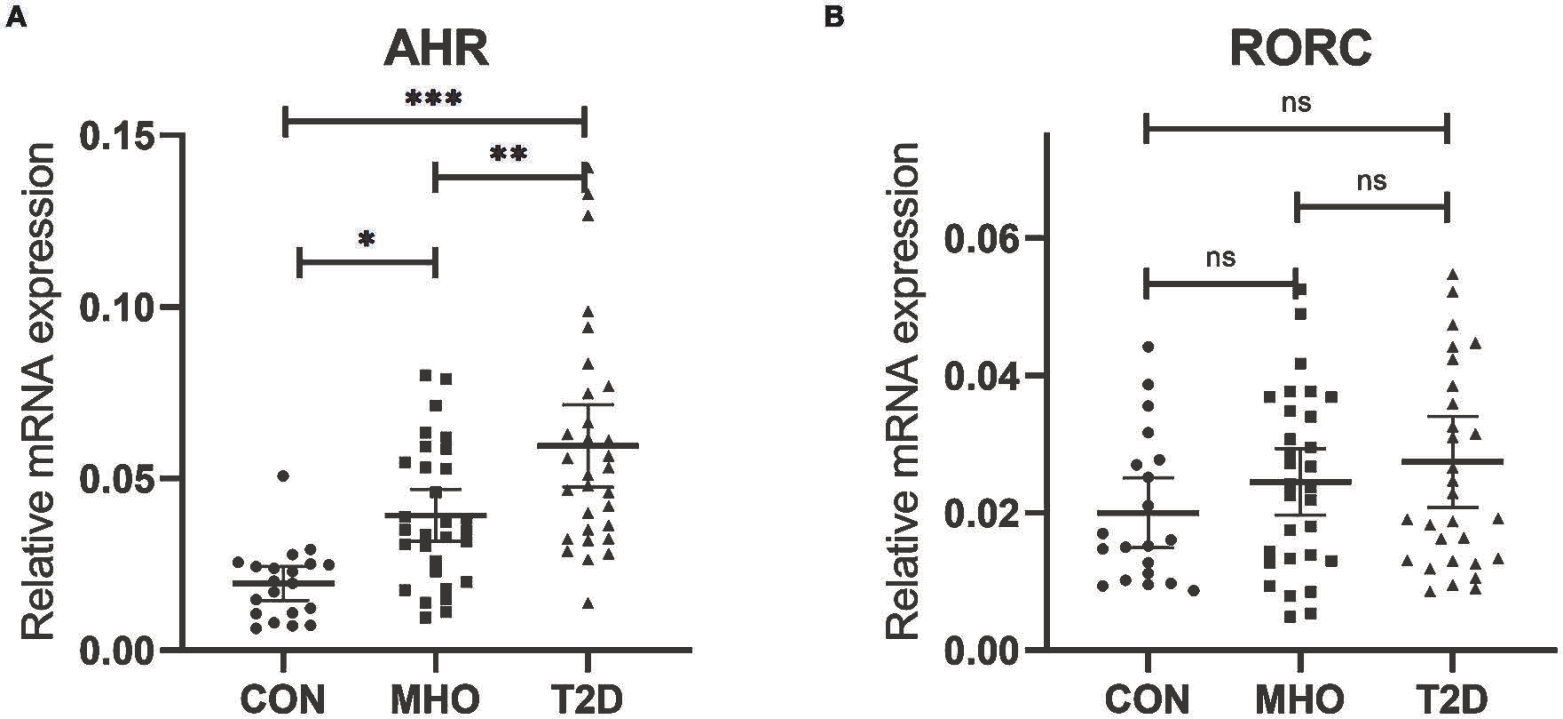
Comparison of Relative mRNA expressions of AHR and RORC. Relative mRNA expressions of AHR **(A)** and RORC **(B)** genes in PBMC (expressed as ratio to GAPDH transcripts). Statistical analysis of the differences between each two groups was performed by Newman–Keuls multiple comparison tests (q-test). *P* < 0.05 were considered significant. *P < 0.05, **P < 0.01, ***P < 0.001.

Previously, we reported an significant increase in peripheral Th22 cells together with Th1 and Th17 cells in MHO and T2D patients. There were also significant higher levels of predominant cytokines in these subjects. Correlation analysis was also performed in all subcohorts of plasma donors and the agreement of elevated Th17, Th22 frequencies with IL-17 and IL-22 was further verified (9). It is well-known that AHR acts as the determinant transcription factor which drives the lineage commitment of Th22 from naïve CD4+ T cells and RORC for Th17. Theoretically, increased peripheral Th22 and Th17 cells could be promoted both/either/neither by AHR and/or RORC signaling in T2D and/or obesity. However, there were no relevant clinical data before. We thus tested whether there were significances of both AHR and RORC expressions (relative to GAPDH) in our case-controlled study. The relative mRNA expressions of AHR (ratio normalized to endogenous control gene) in PBMCs were significantly different among the three subcohorts (^***^P < 0.0001), as shown in Figure 1A. Compared with CTLs (0.0195 ± 0.0106, *n* = 20), the relative mRNA expressions of AHR (compared to GAPDH, same below) was significantly increased, by 2.02- and 3.06-fold respectively, in MHO (0.0394 ± 0.0202, *n* = 30) (^*^P = 0.0145) and T2D (0.0596 ± 0.0322, *n* = 30) (^***^P < 0.0001) patients. An even higher mRNA expressions of AHR were observed in T2D compared with MHO subjects (^**^P = 0.0045).

However, no significant difference was found in the relative mRNA expressions of RORC among the three subcohorts (*P* = 0.2138). The average relative mRNA expressions of RORC seemed higher in PBMCs of MHO (0.0246 ± 0.0108, *n* = 30) and T2D patients (0.0275 ± 0.0129, *n* = 30) compared with CTLs (0.0201 ± 0.0177, *n* = 20). But our present research failed to show statistically-significant difference (Figure 1B).

### Increased AHR mRNA Expressions Correlated With BMI in PBMCs From Non-diabetic Participants

There were no data concerning whether demographic characteristics e.g., age, sex, BMI, and fasting glucose had an influence on the level of AHR expression. Bivariate correlation analysis was performed to check whether the relative AHR expression level had any statistic connection with age, BMI, and fasting blood glucose (FBG). As shown in Table 2, a significantly positive correlation of relative AHR expression was only seen with FBG (*r* = 0.5015, ^*^P < 0.0001, *n* = 80, Spearman analysis) in all the subcohorts. In our present study, however, AHR mRNA expression level showed no statistical correlation with age or BMI. Likewise, RORC mRNA expression level did not have statistical correlation with age, BMI or FBG (shown in Table 3).

**Table 2:** Bivariate correlation analysis of AHR expression with demographic characteristics.

**AHR**	**Age**	**BMI**	**FBG**	**BMI1**	**FBG1**
*n*	80	80	80	50	50
*r* value	0.0875	0.02768	0.5015	0.4783	0.1868
95% confidence interval	−0.1348–0.3014	−0.1932–0.2459	0.3167–0.6496	0.2306–0.6677	−0.0966–0.4422
*P* value (two-tailed)	0.4403	0.807	<0.0001^***^	0.0004^***^	0.1940

BMI1 and FBG1 represent analyses conducted in a subcohort of non-diabetic participants (n = 50) in our study. ^*^ P < 0.05, ^**^ P < 0.01,

*** *P < 0.001*.

**Table 3:** Bivariate correlation analysis of RORC expression with demographic characteristics.

**RORC**	**Age**	**BMI**	**FBG**	**BMI1**	**FBG1**
*n*	80	80	80	50	50
*r*-value	−0.0704	−0.0660	0.1133	0.1638	0.3486
95% confidence interval	−0.2857–0.1517	−0.2816–0.1560	−0.1092–0.3249	−0.1200–0.4229	0.07777–0.5715
*P*-value (two-tailed)	0.5350	0.5610	0.3172	0.2556	0.0131^*^

BMI1 and FBG1 represent analyses conducted in a subcohort of non-diabetic participants (n = 50) in our study.

* P < 0.05, **P < 0.01, ***P < 0.001.

Since the confounding factors such as glucose control and hypoglycemic treatment affect body weight and the FBG level directly, inclusion of T2D subcohort may exert more misunderstanding rather than objective information. We carried out the analyses excluding data from T2D patients (seen in Table 2. BMI1 and FBG1). Not surprisingly, the results were quite opposite. This time the relative AHR mRNA expression level showed no statistical correlation with FBG (*r* = 0.1868, *P* = 0.1940, *n* = 50, Pearson analysis) but had significant and remarkable positive correlation with BMI (*r* = 0.4783, ^***^P = 0.0004, *n* = 50, Pearson analysis). The relative RORC mRNA expression level did not have statistical correlation with age or BMI, but did correlate positively with FBG (*r* = 0.3486, ^*^P = 0.0131, *n* = 50, Pearson analysis) in PBMCs from non-diabetic participants (shown in Table 3). In an attempt to identify the significance and dependency/independence of the association, multiple linear regression analysis was conducted. The well-established inflammatory marker, CRP, was specified as dependent variable in the model. According to preliminary correlation analyses, AHR, together with BMI, TNFα, IL-17, IL-22, and Leptin were entered as independent variables. AHR transcript (relative to that of GAPDH) turned out to be the only independent risk factor of increased CRP levels [β Coefficient: 55.04(18.65–91.43), *P* = 0.005^**^], even after adjusting sex, age, disease status (diabetes duration, and medications).

### Increased Expressions of AHR Gene Correlated With Pro-inflammatory Polarization of Th Lymphocytes in Obesity and T2D Patients

**Figure 2:**
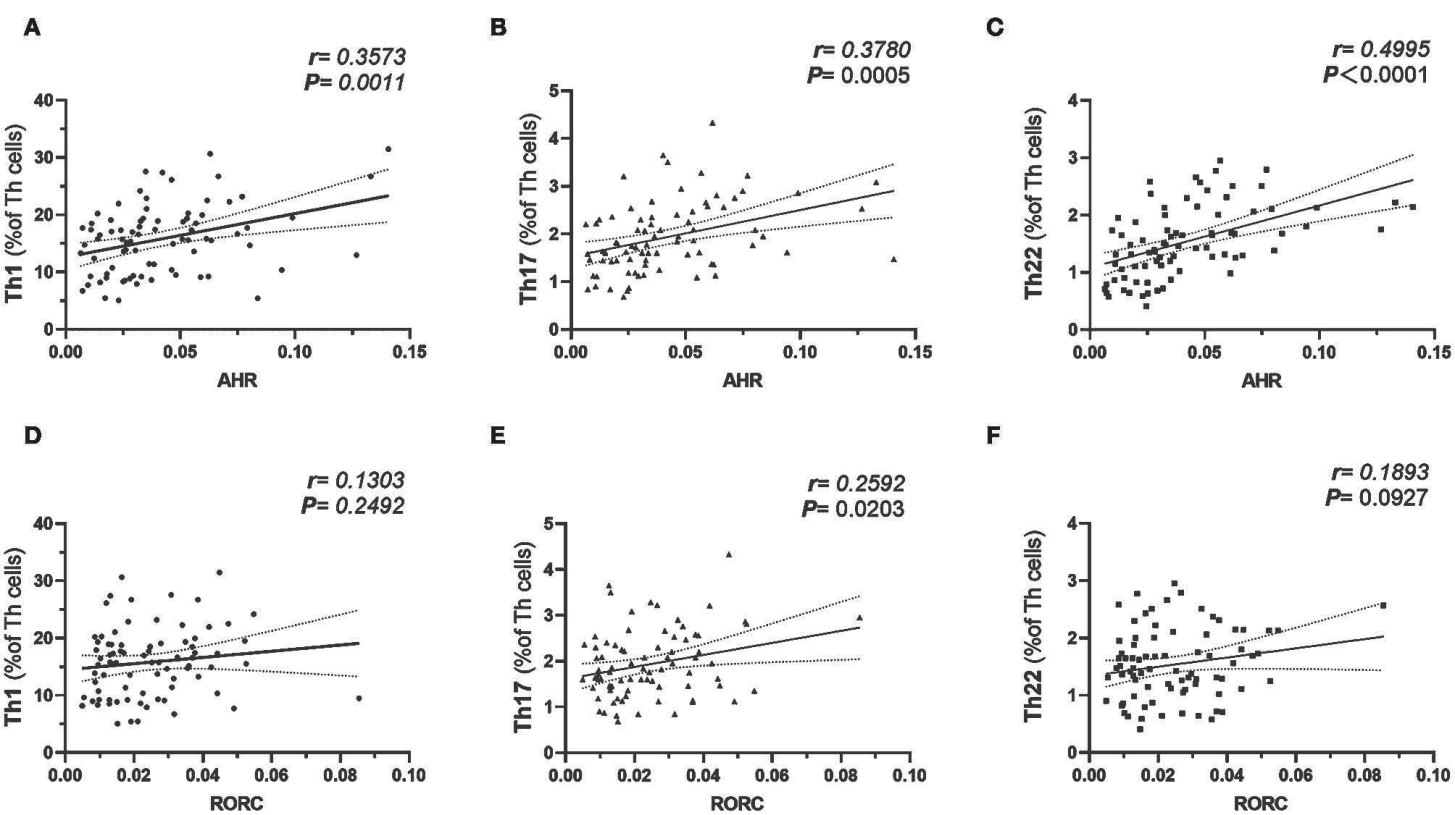
Pearson's correlation analysis between AHR Transcripts (relative to GAPDH) and corresponding peripheral frequencies of Th1 **(A)**, Th17 **(B)**, Th22 **(C)** subsets. Pearson's correlation analysis between RORC Transcripts (relative to GAPDH) and corresponding peripheral frequencies of Th1 **(D)**, Th17 **(E)**, Th22 **(F)** subsets. *r* represents Pearson's correlation coefficient; *P* represents statistical significance.

The plausible links of increased expressions of AHR and RORC with pro-inflammatory Th subsets were verified by previous *in vitro* studies or data from animal models. However, there is still a lack of clinical data regarding whether expressions of the transcription factors correlate with frequencies of corresponding Th subsets in obesity and T2D patients. Notably, AHR also played a role in Th17 differentiation (20). For further analysis of the relationship between the expression of transcription factors and corresponding Th cells frequencies, a correlation study was performed in all subcohorts of participants (*n* = 80). In this study, both AHR and RORC transcript ratios correlated significantly with their corresponding Th subsets. Increased expressions of AHR mRNA matched significantly with elevated Th22 frequency (*r* = 0.4995, Pearson analysis, ^***^P < 0.0001) (Figure 2C). Increased RORC transcripts also matched significantly with Th17 frequency (*r* = 0.2592, Pearson analysis, ^*^P = 0.0203) (Figure 2E). What's more, unanticipated correlations of AHR transcripts with Th1 (*r* = 0.3573, Pearson analysis, ^**^P = 0.0011) and Th17(*r* = 0.3780, Pearson analysis, ^**^P = 0.0005) (Figures 2A,B) were revealed in our study. Although previous studies have indicated a role of AHR signaling in Th17 differentiation, there was no data showing its clinical relevancy with peripheral Th17 frequency in diabetes or obesity. Even fewer studies demonstrated the role of AHR in Th1 polarization. Our data indicated that a general increase in expression of the AHR gene in PBMC not only correlated with the corresponding Th22 lineage, but also correlated with overall pro-inflammatory polarization of other Th subsets in obesity and T2D patients as well. In contrast, correlation of RORC transcripts with Th1 and Th22 might show a remarkable tendency but did not have statistical significance in this study (shown in Figures 2D,F).

### Increased Expressions of AHR Gene Correlated With Elevated Serum Pro-inflammatory Cytokines in Obesity and T2D Patients

**Figure 3:**
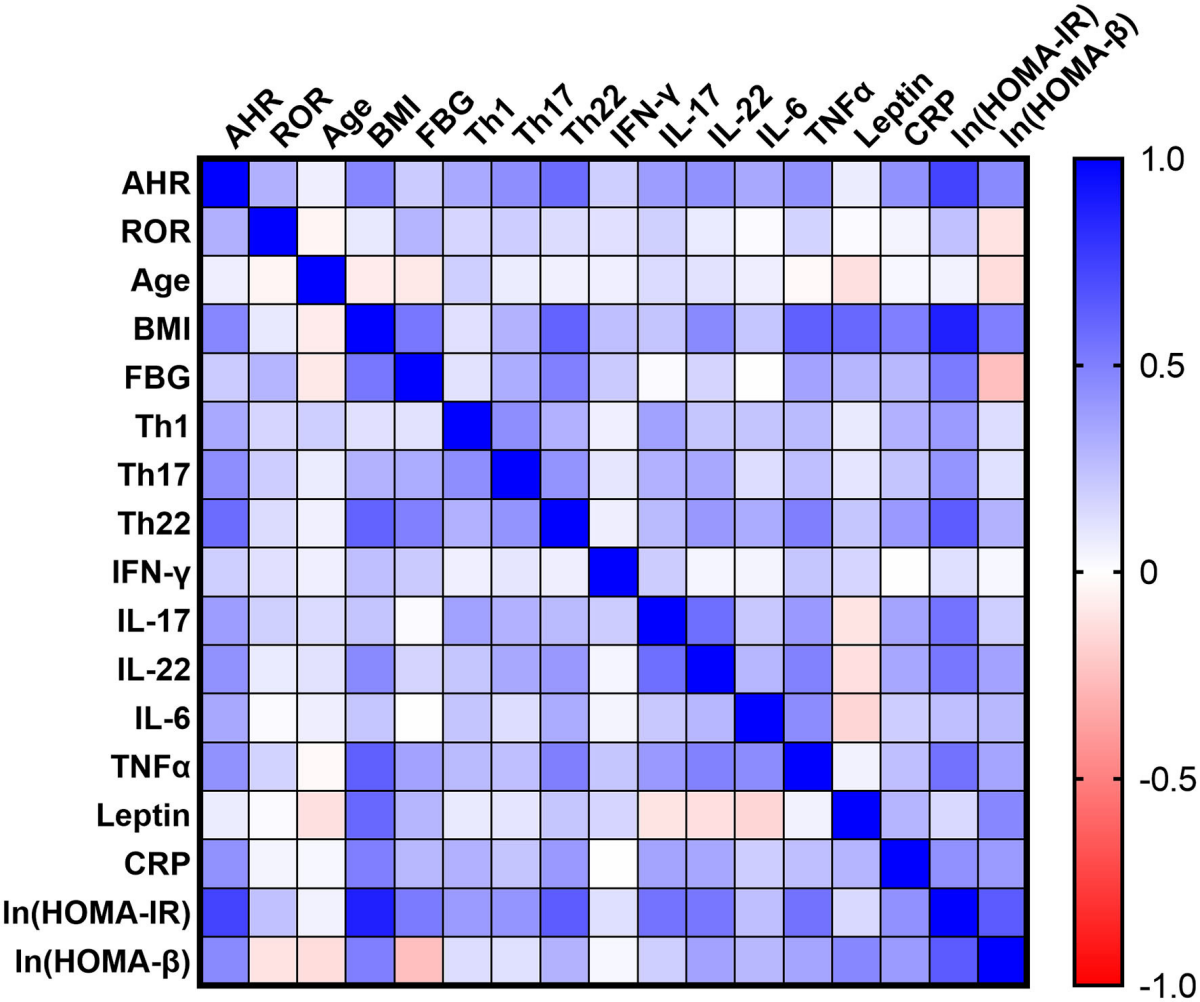
Correlation Matrix: Correlation Coefficients between Each Pair of Parameters. Spearman correlation coefficients between two variables are shown in the heat-map. The correlation coefficients are represented in terms of change of the intensity of red (for negative correlation)/blue (for positive correlation) color, as shown in the color bar. The Spearman correlation coefficients and significances are listed in Supplementary Table 1, and the partial correlation coefficients and significances adjusted by age and sex are also listed in Supplementary Table 2.

To further evaluate the potential role of AHR or RORC in the aberrant Th function, we also assessed the relationship between AHR or RORC transcripts and the downstream pro-inflammatory cytokines in all subcohorts of participants (*n* = 80). Correlation analysis revealed significant relevancies of AHR transcripts with levels of pro-inflammatory cytokines, including IL-17 (*r* = 0.3804, ^**^P = 0.0005, spearman analysis) IL-22 (*r* = 0.4256, ^***^P < 0.0001, spearman analysis) as well as TNFα (*r* = 0.4266, ^***^P < 0.0001, spearman analysis) and IL-6 (*r* = 0.3396, ^*^P = 0.0021, spearman analysis) levels. There was also a remarkable, though statistically non-significant, relevancy with IFN-γ levels (*r* = 0.1853, *P* = 0.0999, spearman analysis) (shown in Figure 3 and Supplementary Table 1). hsCRP is a well-established marker for the meta-inflammatory status, especially in obesity and diabetes. Here we also found a significantly positive correlation of AHR transcripts with serum hsCRP levels (*r* = 0.4244, ^***^P < 0.0001, spearman analysis). To note, Th17, whose differentiation is driven by ROR signaling, represents another important source of IL-22. However, we failed to find statistically significant relevance between expression of RORC with above mentioned cytokines or markers (shown in Figure 3). Together with the correlation analysis of Th lymphocyte polarization, our data indicate that AHR, rather than ROR signaling, might have closer ties with the pro-inflammatory status in T2D and obesity.

Among the emerging mediators of inflammation, leptin, a key controller of metabolic function, has been extensively implicated in modulating immune responses and promoting chronic inflammatory responses in peripheral tissues. In our cohort, serum leptin levels also correlated significantly with BMI (*r* = 0.5887, ^***^P < 0.0001, spearman analysis), FBG (*r* = 0.2862, ^*^P = 0.0439, spearman analysis) (*n* = 50, as T2D group was excluded concerning BMI and FBG as mentioned above) and hsCRP (*r* = 0.2912, ^*^P = 0.0088, spearman analysis). However, the present data did not establish significant correlation of AHR (*r* = 0.0709, *P* = 0.5321, spearman analysis) or RORC transcripts (*r* = 0.01201, *P* = 0.9157, spearman analysis) with serum leptin levels (shown in Figure 3).

### Elevated AHR Expression Positively Correlated With Both Insulin Resistance and Islets β-Cell Function

**Figure 4:**
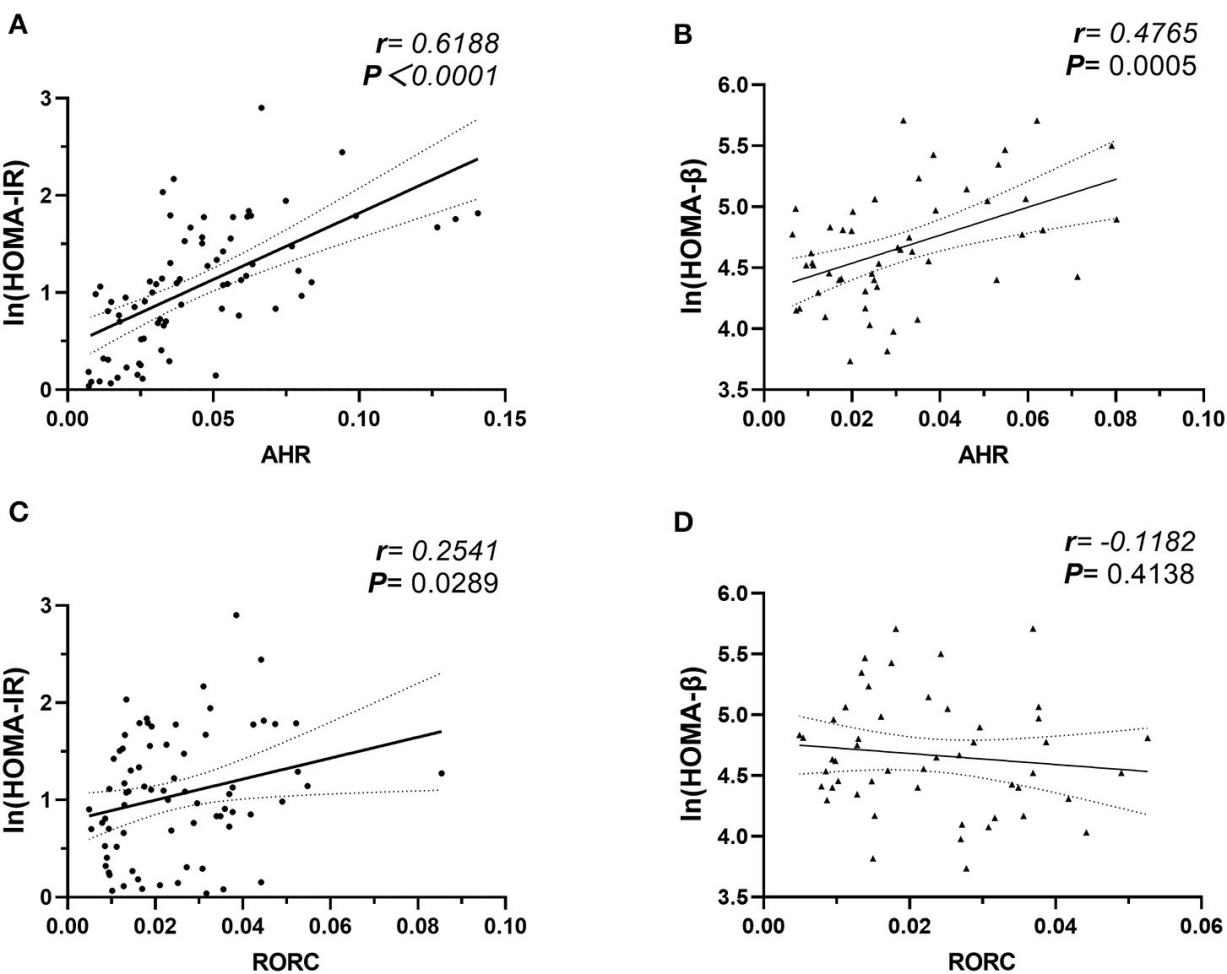
Correlation Analysis of AHR and RORC Transcripts with Ln (HOMA-IR) and Ln (HOMA-β). Pearson's correlation analysis between AHR and RORC Transcripts (relative to GAPDH) and Ln (HOMA) parameters. Note that correlation of AHR **(A)** and RORC **(C)** transcripts with Ln (HOMA-IR) values were conducted in all participants (*n* = 80), while correlation of AHR **(B)** and RORC **(D)** transcripts with Ln (HOMA-β) values were conducted in subcohorts of CTL and MHO (*n* = 50), respectively.

We further studied the potential role of elevated AHR mRNA expression in the development of insulin resistance. In our study, the correlation analysis revealed notable relevance of both elevated AHR (*r* = 0.6188, ^***^P < 0.0001, Pearson analysis) and RORC (*r* = 0.2541, ^*^P = 0.0289, Pearson analysis) (relative to GAPDH) mRNA expression to increased insulin resistance (Figure 4). We further conducted multiple linear regression analysis to rule out potential confounders in association with HOMA index for insulin resistance. AHR, together with RORC, BMI, TNFα, IL-6, IL-17, IL-22, and CRP were entered as independent variables. AHR transcript (relative to that of GAPDH) turned out to be an independent risk factor of increased CRP levels [β Coefficient: 9.25(0.46–18.03), *P* = 0.040^*^], even after adjusting sex, age, disease status (diabetes duration and medications).

As an even more significant increase of AHR transcripts was found in T2D patients compared with MHO, we tested the assumption that whether the increase of AHR transcripts in PBMCs correlated with the compensation of islet β-cell function. Since we did not request our patients to stop necessary hypoglycemic treatment (especially insulin preparations and secretagogues) before sampling, the treatment had potential neglectable influences in insulin levels. The insulin levels in these patients could not reflect the intrinsic β-cell function. Therefore, the data from T2D groups were excluded in this analysis. In subcohorts of CTL and MHO (*n* = 50), our research demonstrated an unexpected significant positive correlation between AHR transcripts and Ln (HOMA-β) values (*r* = 0.4765, ^**^P = 0.0005, Pearson analysis) (see Figure 4). No significant correlation was identified between Ln (HOMA-β) and that of RORC (*r* = −0.1182, *P* = 0.4138, Pearson analysis). Moreover, in a multiple linear regression model with AHR, BMI, TNFα, IL-17, IL-22, leptin, and CRP included as independent variables, AHR transcript (relative to that of GAPDH) also represented as an independent predictive factor of increased Ln (HOMA-β) (β Coefficient: 9.25(0.46–18.03), *P* = 0.040^*^) in non-diabetic participants, even after adjusting age and sex.

## Discussion

Obese individuals are typically accompanied by deleterious metabolic profiles, especially Type 2 diabetes. But various epidemiological studies have identified a subset of patients with a normal metabolic phenotype referred to as the metabolically healthy obesity (MHO) (29). MHO is a novel concept that stratifies obese individuals according to their metabolic status (30). Studies into MHO provide important implications for both understanding of the disease progression and the targeted prevention of obesity related metabolic disorders (31). However, there are conflicting evidences in the literature on this topic regarding its risk profile. But generally, MHO show a substantially increased risk of developing T2D compared with metabolically healthy normal-weight adults (CTL) (32). Insulin resistance is well-known as the dominant initiating and persisting factor in obesity-associated metabolic disorders, and decompensated β-cell function is the determinant process distinguishing T2D from normoglycemic obesity (33). However, the culprits for the deterioration and decompensation have not been identified. In the past two decades, the chronic low-grade inflammation, referred to as meta-inflammation, has been established as the central link between obesity and T2D (3–5). Moreover, emerging data revealed that meta-inflammation was also present in MHO so that the “metabolically healthy” might not represent a persistent favorable condition (4, 9, 29). However, more studies are needed to evaluate the meta-inflammation in obesity from two separate subcohorts (34). This study was designed to include both T2D patients and MHO subjects, and thus demonstrated more similarities as well as distinguishment between MHO and T2D.

Our study revealed significant elevation in Ln (HOMA-IR) and serum levels of leptin, insulin, TNFα, IL-22, and hsCRP in MHO compared with CTLs. The data indicated that obesity, either metabolically healthy or not, was accompanied with hyperinsulinemia, leptinemia, and low-grade inflammation. Serum levels of pro-inflammatory cytokines including IL-17, IL-22, and IL-6 were even significantly higher in T2D patients compared with the MHO group, indicating an aggravated inflammatory status. The persistent and progressive status of meta-inflammation accompanies the whole trajectory of the two diseases, from its pathogenesis to complication development (35). Diabetic patients show an dysregulated number and function of immune cells, of both innate and adaptive immunity (4). Accumulating evidence established that immune cells especially T lymphocyte alterations preceded the loss of insulin sensitivity in adipose tissue and contribute to the general pro-inflammatory drift observed in obesity and T2D (4, 7). Accumulating evidence identified an elevation of pro-inflammatory subsets together with a significant decrease in Tregs, which may directly evoke hyperactivation of innate immunity and then meta-inflammation, which contributes greatly to both insulin resistance and β-cell damage (10–14). More thorough investigations are disclosing the possible “triggers” of inflammatory responses, most of which are at the crossroad of environmental signaling, metabolic sensing and immune modulation, e.g., leptin (36). More recently, data on how pro-inflammatory Th cells and/or effector cytokines affect energy metabolism have been piling up. However, the primary adapter which senses the environmental signals (e.g., dietary components (37), daily schedule, gut microbiota, environmental pollutants and endogenous metabolites etc.) and decodes them into metabolic and immunologic disturbance still remains ill-defined.

A biological entity that tightly links genes and the environment is AHR, which is a nuclear receptor best known for its role in xenobiotic metabolism. AHR is an ancient transcription factor which was first identified with its role in modulating the organism's response to xenobiotics, e.g., polycyclic aromatic hydrocarbons (15). Previous work establishes AHR as a pivotal environmental modifier that integrates signals from chemical exposure in the regulation of lipid and energy metabolism (16). With advances in the knowledge of AHR physiology, constitutive AHR signaling driven by both environmental signals and endogenous metabolites has been deemed to be of crucial importance for the maintenance of AHR-governed cellular processes, including hormone levels, circadian clock, gastrointestinal homeostasis, immune regulation and cell proliferation (16, 17). Upon ligand binding, the AHR translocates to the nucleus, where it binds to forms a complex with the AHR nuclear translocator (ARNT). The AHR/ARNT heterodimer regulates the transcription of genes in the cytochrome P450 Cyp1 family and thousands of other genes (24) including other nuclear receptors relevant to obesity (38).

Moreover, in the past decade, AHR has been increasingly recognized as an important modulator in immune and inflammatory responses (18). AHR has been proved to be the determinant transcription factor driving the development of Th22 (19). It has also been shown to regulate the differential programs of Tregs and Th17 cells (20). Moreover, AHR signaling in other immune cell populations, e.g., monocytes, also contributed to priming CD4+ T cells to skewed differentiation (39). Our previous work added weight to the evidence that Th cell polarization contributed to the pathogenesis of obesity and T2D. Apart from evidence from animal models, correlation between pro-inflammatory Th cells and metabolic parameters implied that the imbalance of T cell subsets was responsible for the pathogenesis of obesity and T2D in human (9, 14). We and others also identified significantly elevated Th22 frequencies from peripheral blood of both MHO and T2D patients, suggesting the possible role of Th22 subsets in disease progression (9, 11). However, there was no human study present concerning the potential triggers driving the pro-inflammatory polarization of Th subsets. In this study, the relative amount of transcripts of the transcription factors AHR and RORC in PBMCs responsible for Th17 and Th22 differentiation were determined in obesity and T2D patients. Our study first identified that AHR transcripts (relative to GAPDH) were notably increased in the PBMCs from both obesity and T2D patients compared with lean healthy subjects with a normal BMI. Elevation of AHR mRNA expression was even more remarkable in PBMCs of patients with T2D compared to MHO patients. However, no significant difference was found in the transcripts of RORC among the three subcohorts. We further evaluated whether there was correlation between the two transcription factors with the demographic or basic clinical parameters. AHR transcripts only showed a significant and remarkable positive correlation with BMI in the subcohorts of non-diabetic participants. RORC transcripts did not have statistical correlation with age or BMI but did correlate positively with FBG in PBMCs from non-diabetic participants. These data primarily established a link of AHR and RORC expression with obesity and glucose intolerance. And the negative findings between demographic parameters and AHR and RORC expression also helped to distinguish the confounding factors in the further analysis between subcohorts. Both AHR and RORC transcripts correlated significantly with their corresponding Th subsets, verifying the agreement of expressions of transcription factors in PBMCs with corresponding Th cells frequencies. Since AHR is well-established as the major driver of Th22 commitment, our data explained at least partially for the increased Th22 cells in obesity and T2D. Moreover, unanticipated correlations among AHR transcripts and Th1 and Th17 cells were revealed in our further analysis, indicating that increased expressions of AHR gene may contribute to the overall pro-inflammatory polarization of Th lymphocytes in obesity and T2D patients. This observational study only revealed statistical correlations but could not support a causal relationship. However, considering its well-established transcription activity, AHR would more likely to be an important regulator of immune response (18), rather than consequence of Th polarization in MHO and T2D (40). AHR expression in PBMCs would more likely to be a causative factor in Th polarization, even though the intermediate links and mechanisms are currently unknown. It is important to note that the expressions of the transcription factors were determined in PBMCs but not isolated T lymphocytes in this study. Changes in the expressions of transcription factors in PBMCs may be not only associated with changes in Th cells, since other cell subpopulations, such as monocytes, also demonstrate considerable AHR expression. Given that AHR may not be involved in Th1 polarization, it is quite possible that Th polarization in obesity and T2D may be regulated, at least partially, by expressions of AHR in other cell populations (e.g., monocyte) other than CD4+ T lymphocyte itself. In contrast, correlation of RORC transcripts with Th1 and Th22 might show a remarkable tendency but did not have statistical significance in our study. To further evaluate the potential role of AHR or RORC in the aberrant Th cell function, we also assessed the relationship between AHR or RORC transcripts and the corresponding cytokines in plasma of participants of all subcohorts. The present data revealed remarkable relevancies of AHR transcripts with not only IL-22, but also IFN-γ, IL-17 as well as TNFα and IL-6 levels. To note, Th17, whose differentiation is driven by ROR signaling, represents another important source of IL-22 (19). However, we failed to find statistically significant relevance between expressions of RORC with relevant cytokines or markers. Taken together, our data indicate that AHR, rather than ROR signaling, might have a more important role in the pro-inflammatory status in individuals with T2D and obesity. With a significant positively correlation with serum hsCRP levels, AHR transcripts in PBMCs may represent as a novel mediator of the immune disturbance and meta-inflammation in obesity and T2D patients, independent of leptin, the previously-known key metabolic controller and immune modulator (36). What's more, it is quite possible that the overall pro-inflammatory status in T2D and MHO may be associated to a general increase in AHR levels in PBMCs, which is at least partially, independent from Th cell polarization.

Up to now, there is a growing body of evidence concerning the role of the AHR signaling in obesity and T2D. Studies have shown that the toxicant-activated AHR may disrupt fat metabolism and contribute to obesity. Adipocyte AHR has been proved responsible for pollutants (e.g., biphenyls) induced adipose inflammation and impairment of glucose homeostasis in mice (41). AHR overactivation directly promotes obesity, hepatic steatosis and insulin resistance under HFD exposure (21, 24). More recently, AHR was even proven to be the major adapter integrating signals from gut microbiota alternation (42) and circadian clock disruption (21) with metabolic dysfunction. Taken together, AHR signaling established a critical role in obesity and glucose intolerance in animal models. Our work provided clinical data verifying the alternations in AHR expression in PBMCs of both patients with obesity and T2D patients. We further studied the possible role of elevated AHR transcripts in the pathogenesis of insulin resistance. The present data showed a remarkable linear correlation of AHR transcripts with Ln (HOMA-IR), indicating that AHR signaling may contribute greatly to insulin resistance. What's more, an unanticipated positive relevancy of AHR transcripts with basal β-cell function was revealed. Different with previous work demonstrating a remarkable negative correlation between Th22 frequencies and Ln (HOMA-β) in drug-naïve T2D patients (9), the present data showed a positive correlation of AHR transcripts with Ln (HOMA-β) in non-diabetic participants. Previous study indicated peripheral Th22 cell frequencies might be associated with decompensation of β cell function in T2D patients. However, in this study, the correlation of AHR transcripts with Ln (HOMA-β) may indicated a possible role of compensated insulin secretion in health donors and more especially in MHO individuals. These findings may not support the contention that the possible influence of AHR expressions in PBMC on β cell compensation or dysfunction in obesity and T2D depend totally on Th22 cells. Moreover, IL-22 secreting ILC3s, whose differentiation was also driven by AHR transcription activity, were proved more recently to be potentially protective in maintaining immunologic and metabolic homeostasis (43, 44). However, circulating IL-22 producing Th subsets or even neutrophils had deteriorating role in immunologic and metabolic disturbance (45). As reported in animal models, AHR signaling, together with IL-22, may exert a double-sided effect in β-cell dysfunction and compensation (44, 46). Therefore, intrinsic increase in AHR expression, together with environmental factors influencing AHR signaling (such as endogenous or exogenous ligands), may exert multifaceted influence in the pathophysiology of the metabolic disorders.

Collectively, we identified for the first time significant elevated AHR transcripts from peripheral blood of both MHO and T2D patients. Increased expressions of the AHR gene may contribute to the overall pro-inflammatory polarization of Th lymphocytes in obesity and T2D patients. Our data preliminarily suggest that AHR signaling might have a dual role in both development of insulin resistance and compensated β-cells function. In context of the emerging multidisciplinary research about AHR signaling, further understanding of the mechanisms underlying may provide a novel therapeutic target for obesity and its related metabolic disorders.

## Data Availability Statement

Access to more primary data will be considered by the author upon request ( rusingstar@163.com). The authors will make the primary data available under the premise of privacy protection, restricted usage, and duplication.

## Ethics Statement

The studies involving human participants were reviewed and approved by Ethics committee of Qilu Hospital of Shandong University. The patients/participants provided their written informed consent to participate in this study.

## Author Contributions

RZ, XH, and LC contributed conception and design of the study. RZ, SS, QH, JQ, PL, and YS conducted the investigation. RZ, SS, QH, and LS organized the database. RZ, LS, and JS performed the statistical analysis. RZ wrote the first draft of the manuscript. SS, JQ, QH, YS, and PL wrote sections of the manuscript. All authors contributed to manuscript revision, read, and approved the submitted version.

## Conflict of Interest

The authors declare that the research was conducted in the absence of any commercial or financial relationships that could be construed as a potential conflict of interest.

## References

[1] Unnikrishnan R Pradeepa R Joshi SR Mohan V. Type 2 diabetes: demystifying the global epidemic. Diabetes. (2017) 66:1432–42. 10.2337/db16-0766 28533294

[2] Flier JS. Obesity wars: molecular progress confronts an expanding epidemic. Cell. (2004) 116:337–50. 10.1016/S0092-8674(03)01081-X 14744442

[3] Kanneganti TD Dixit VD Immunological complications of obesity. Nat Immunol. (2012) 13:707–12. 10.1038/ni.2343 22814340

[4] Shu CJ Benoist C Mathis D. The immune system's involvement in obesity-driven type 2 diabetes. Semin Immunol. (2012) 24:436–42. 10.1016/j.smim.2012.12.001 23333525 PMC3582811

[5] Ndisang JF Rastogi S Vannacci A. Immune and inflammatory processes in obesity, insulin resistance, diabetes, and related cardiometabolic complications. J Immunol Res. (2014) 2014:579560. 10.1155/2014/579560 25328894 PMC4189852

[6] Zhao RX Li WJ Lu YR Qin J Wu CL Tian M. Increased peripheral proinflammatory T helper subsets contribute to cardiovascular complications in diabetic patients. Mediat Inflamm. (2014) 2014:596967. 10.1155/2014/596967 24803740 PMC3997161

[7] Lumeng CN Maillard I Saltiel AR. T-ing up inflammation in fat. Nat Med. (2009) 15:846–7. 10.1038/nm0809-846 19661987

[8] Yang H Youm YH Vandanmagsar B Ravussin A Gimble JM Greenway F. Obesity increases the production of proinflammatory mediators from adipose tissue T cells and compromises TCR repertoire diversity: implications for systemic inflammation and insulin resistance. J Immunol. (2010) 185:1836–45. 10.4049/jimmunol.1000021 20581149 PMC4829921

[9] Zhao R Tang D Yi S Li W Wu C Lu Y. Elevated peripheral frequencies of Th22 cells: a novel potent participant in obesity and type 2 diabetes. PloS ONE. (2014) 9:e85770. 10.1371/journal.pone.0085770 24465695 PMC3894984

[10] Xu X Zheng S Yang F Shi Y Gu Y Chen H. Increased Th22 cells are independently associated with Th17 cells in type 1 diabetes. Endocrine. (2013) 46:90–8. 10.1007/s12020-013-0030-z 23928796

[11] Dalmas E Venteclef N Caer C Poitou C Cremer I Aron-Wisnewsky J. T cell-derived IL-22 amplifies IL-1beta-driven inflammation in human adipose tissue: relevance to obesity and type 2 diabetes. Diabetes. (2014) 63:1966–77. 10.2337/db13-1511 24520123

[12] Fabbrini E Cella M McCartney SA Fuchs A Abumrad NA Pietka TA. Association between specific adipose tissue CD4+ T-cell populations and insulin resistance in obese individuals. Gastroenterology. (2013) 145:366–74 e1–3. 10.1053/j.gastro.2013.04.010 23597726 PMC3756481

[13] Jagannathan-Bogdan M McDonnell ME Shin H Rehman Q Hasturk H Apovian CM. Elevated proinflammatory cytokine production by a skewed T cell compartment requires monocytes and promotes inflammation in type 2 diabetes. J Immunol. (2011) 186:1162–72. 10.4049/jimmunol.1002615 21169542 PMC3089774

[14] Zeng C Shi X Zhang B Liu H Zhang L Ding W. The imbalance of Th17/Th1/Tregs in patients with type 2 diabetes: relationship with metabolic factors and complications. J Mol Med. (2012) 90:175–86. 10.1007/s00109-011-0816-5 21964948

[15] Okey AB Riddick DS Harper PA. Molecular biology of the aromatic hydrocarbon (dioxin) receptor. Trends Pharmacol Sci. (1994) 15:226–32. 10.1016/0165-6147(94)90316-6 7940984

[16] Denison MS Nagy SR. Activation of the aryl hydrocarbon receptor by structurally diverse exogenous and endogenous chemicals. Ann Rev Pharmacol Toxicol. (2003) 43:309–34. 10.1146/annurev.pharmtox.43.100901.135828 12540743

[17] Bock KW. Aryl hydrocarbon receptor (AHR): from selected human target genes and crosstalk with transcription factors to multiple AHR functions. Biochem Pharmacol. (2019) 168:65–70. 10.1016/j.bcp.2019.06.015 31228464

[18] Gutierrez-Vazquez C Quintana FJ. Regulation of the immune response by the aryl hydrocarbon receptor. Immunity. (2018) 48:19–33. 10.1016/j.immuni.2017.12.012 29343438 PMC5777317

[19] Ramirez JM Brembilla NC Sorg O Chicheportiche R Matthes T Dayer JM. Activation of the aryl hydrocarbon receptor reveals distinct requirements for IL-22 and IL-17 production by human T helper cells. Eur J Immunol. (2010) 40:2450–9. 10.1002/eji.201040461 20706985

[20] Hao N Whitelaw ML. The emerging roles of AhR in physiology and immunity. Biochem Pharmacol. (2013) 86:561–70. 10.1016/j.bcp.2013.07.004 23856287

[21] Xu CX Wang C Zhang ZM Jaeger CD Krager SL Bottum KM. Aryl hydrocarbon receptor deficiency protects mice from diet-induced adiposity and metabolic disorders through increased energy expenditure. Int J Obes. (2015) 39:1300–09. 10.1038/ijo.2015.63 25907315 PMC4526411

[22] Fujiyoshi PT Michalek JE Matsumura F. Molecular epidemiologic evidence for diabetogenic effects of dioxin exposure in U.S. Air force veterans of the Vietnam war. Environ Health Perspect. (2006) 114:1677–83. 10.1289/ehp.9262 17107852 PMC1665440

[23] Rajagopalan S Brook RD. Air pollution and type 2 diabetes: mechanistic insights. Diabetes. (2012) 61:3037–45. 10.2337/db12-0190 23172950 PMC3501850

[24] Kerley-Hamilton JS Trask HW Ridley CJ Dufour E Ringelberg CS Nurinova N. Obesity is mediated by differential aryl hydrocarbon receptor signaling in mice fed a Western diet. Environ Health Perspect. (2012) 120:1252–9. 10.1289/ehp.1205003 22609946 PMC3440132

[25] Zou J Chassaing B Singh V Pellizzon M Ricci M Fythe MD. Fiber-mediated nourishment of gut microbiota protects against diet-induced obesity by restoring IL-22-mediated colonic health. Cell Host Microbe. (2018) 23:41–53 e4. 10.1016/j.chom.2017.11.003 29276170 PMC6005180

[26] Cheng HY Ning MX Chen DK Ma WT. Interactions between the gut microbiota and the host innate immune response against pathogens. Front Immunol. (2019) 10:607. 10.3389/fimmu.2019.00607 30984184 PMC6449424

[27] Basu R O'Quinn DB Silberger DJ Schoeb TR Fouser L Ouyang W. Th22 cells are an important source of IL-22 for host protection against enteropathogenic bacteria. Immunity. (2012) 37:1061–75. 10.1016/j.immuni.2012.08.024 23200827 PMC3678257

[28] Haffner SM Miettinen H Stern MP The homeostasis model in the San Antonio Heart Study. Diabetes Care. (1997) 20:1087–92. 10.2337/diacare.20.7.1087 9203442

[29] Smith GI Mittendorfer B Klein S. Metabolically healthy obesity: facts and fantasies. J Clin Investig. (2019) 129:3978–89. 10.1172/JCI129186 31524630 PMC6763224

[30] Beh S. Is metabolically healthy obesity a useful concept? Diabet Med. (2019) 36:539–45. 10.1111/dme.13869 30474298

[31] Iacobini C Pugliese G Blasetti Fantauzzi C Federici M Menini S. Metabolically healthy versus metabolically unhealthy obesity. Metabolism. (2019) 92:51–60. 10.1016/j.metabol.2018.11.009 30458177

[32] Camhi SM Must A Gona PN Hankinson A Odegaard A Reis J. Duration and stability of metabolically healthy obesity over 30 years. Int J Obes. (2019) 43:1803–10. 10.1038/s41366-018-0197-8 30158567 PMC6395568

[33] Boni-Schnetzler M Ehses JA Faulenbach M Donath MY. Insulitis in type 2 diabetes. Diabetes Obes Metabol. (2008) 10(Suppl. 4):201–4. 10.1111/j.1463-1326.2008.00950.x 18834448

[34] Alam I Ng TP Larbi A Does inflammation determine whether obesity is metabolically healthy or unhealthy? The aging perspective. Mediat Inflamm. (2012) 2012:456456 10.1155/2012/456456 PMC3471463 23091306

[35] Donath MY Boni-Schnetzler M Ellingsgaard H Ehses JA. Islet inflammation impairs the pancreatic beta-cell in type 2 diabetes. Physiology. (2009) 24:325–31. 10.1152/physiol.00032.2009 19996363

[36] Matarese G Procaccini C De Rosa V Horvath TL La Cava A. Regulatory T cells in obesity: the leptin connection. Trends Mol Med. (2010) 16:247–56. 10.1016/j.molmed.2010.04.002 20493774

[37] McMillan BJ Bradfield CA. The aryl hydrocarbon receptor is activated by modified low-density lipoprotein. Proc Natl Acad Sci USA. (2007) 104:1412–7. 10.1073/pnas.0607296104 17227852 PMC1783125

[38] Wang C Xu CX Krager SL Bottum KM Liao DF Tischkau SA. Aryl hydrocarbon receptor deficiency enhances insulin sensitivity and reduces PPAR-alpha pathway activity in mice. Environ Health Perspect. (2011) 119:1739–44. 10.1289/ehp.1103593 21849270 PMC3261983

[39] Castaneda AR Pinkerton KE Bein KJ Magana-Mendez A Yang HT Ashwood P. Ambient particulate matter activates the aryl hydrocarbon receptor in dendritic cells and enhances Th17 polarization. Toxicol Lett. (2018) 292:85–96. 10.1016/j.toxlet.2018.04.020 29689377 PMC5971007

[40] Rothhammer V Quintana FJ. The aryl hydrocarbon receptor: an environmental sensor integrating immune responses in health and disease. Nat Rev Immunol. (2019) 19:184–97. 10.1038/s41577-019-0125-8 30718831

[41] Baker NA Shoemaker R English V Larian N Sunkara M Morris AJ. Effects of adipocyte aryl hydrocarbon receptor deficiency on pcb-induced disruption of glucose homeostasis in lean and obese mice. Environ Health Perspect. (2015) 123:944–50. 10.1289/ehp.1408594 25734695 PMC4590748

[42] Delzenne NM Knudsen C Beaumont M Rodriguez J Neyrinck AM Bindels LB. Contribution of the gut microbiota to the regulation of host metabolism and energy balance: a focus on the gut-liver axis. Proc Nutr Soc. (2019) 78:319–28. 10.1017/S0029665118002756 30628563

[43] Diefenbach A Gnafakis S Shomrat O. Innate lymphoid cell-epithelial cell modules sustain intestinal homeostasis. Immunity. (2020) 52:452–63. 10.1016/j.immuni.2020.02.016 32187516

[44] Miani M Le Naour J Waeckel-Enee E Verma SC Straube M Emond P. Gut microbiota-stimulated innate lymphoid cells support beta-defensin 14 expression in pancreatic endocrine cells, preventing autoimmune diabetes. Cell Metabol. (2018) 28:557–72 e6. 10.1016/j.cmet.2018.06.012 30017352

[45] Xue J Zhao Q Sharma V Nguyen LP Lee YN Pham KL. Aryl hydrocarbon receptor ligands in cigarette smoke induce production of interleukin-22 to promote pancreatic fibrosis in models of chronic pancreatitis. Gastroenterology. (2016) 151:1206–17. 10.1053/j.gastro.2016.09.064 27769811 PMC5499510

[46] Natividad JM Agus A Planchais J Lamas B Jarry AC Martin R. Impaired aryl hydrocarbon receptor ligand production by the gut microbiota is a key factor in metabolic syndrome. Cell Metab. (2018) 28:737–49e4. 10.1016/j.cmet.2018.07.001 30057068

