# Supplementary material for: Increased AHR Transcripts Correlate With Pro-inflammatory T-Helper Lymphocytes Polarization in Both Metabolically Healthy Obesity and Type 2 Diabetic Patients
Source: Front Immunol. 2020 Jul 30;11:1644. doi: 10.3389/fimmu.2020.01644 (PMC7406643; doi:10.3389/fimmu.2020.01644)
Supplement: Supplementary file 1 [file Table_1.DOCX]

Supplementary Table 1. Correlation Matrix: Spearman Correlation Coefficients and significances between Each Pair of Parameters.

Supplementary Table 2. Correlation Matrix: Partial Correlation Coefficients and significances between Each Pair of Parameters (adjusted by age and sex).
